# Conserving surgery in inflammatory breast cancer after neoadjuvant chemotherapy in patients with clinical complete response: the ConSIBreC randomized controlled trial

**DOI:** 10.3389/fonc.2024.1372633

**Published:** 2024-02-22

**Authors:** Lorenzo Scardina, Riccardo Masetti, Gianluca Franceschini

**Affiliations:** Multidisciplinary Breast Center, Dipartimento Scienze della Salute della Donna e del Bambino e di Sanità Pubblica, Fondazione Policlinico Universitario Agostino Gemelli IRCCS, Rome, Italy

**Keywords:** inflammatory breast cancer, breast conserving surgery, radical mastectomy, neoadjuvant chemotherapy, ConSIBreC trial

## Background

Inflammatory breast cancer (IBC) is an infrequent, distinct clinicopathologic entity among breast cancer subtypes. It is one of the highly aggressive subtypes and contributes significantly to breast cancer–related mortality.

It affects about 2 - 3% of women diagnosed with breast cancer (1) and approximately 30% of patients have distant metastasis at the time of presentation (2). Clinical outcomes of IBC are poor with median overall five years survival of 55% (3).

IBC is a clinical syndrome in patients with invasive breast cancer and the diagnosis is based upon criteria that include a rapid onset of breast inflammatory signs in less than 6 months, such as erythema, edema and peau d’orange involving more than one-third of the skin (4).

The disease is characterized by florid tumor emboli that obstruct dermal lymphatics, leading to swelling and inflammation of the affected breast. Lymphatic vessel invasion results in a high rate of local failure and a high tendency of visceral metastases in IBC. Although the skin changes of such emboli are histopathologically distinctive, they are neither necessary nor sufficient by themself to make a diagnosis of IBC.

In the 8^th^ edition of the American Joint Committee on Cancer IBC is classified cT4d and patients are considered as having at least stage IIIB disease (5); besides, due to the aggressive nature, a high proportion of IBC presents lymph node involvement (80%) (6).

By its definition, IBC is non-operable at diagnosis. According to National Comprehensive Cancer Network guidelines, neoadjuvant chemotherapy (NAC) is the recommended initial treatment for IBC. If a patient responds well to NAC, radical mastectomy with axillary lymph node dissection and postoperative radiation therapy to the chest wall is commonly performed (7). Further systemic adjuvant therapies will also have to be performed subsequently based on the molecular profile of the tumor and the residual disease after NAC.

However, thanks to the increased rate of pathological complete response after NAC, there is an emerging question whether it is possible to perform a breast conserving surgery in IBC patients.

According to the UK IBC working group, breast conserving surgery in excellent responders can be considered and there is no justification for the mandatory requirement for maintaining the convention of recommending mastectomy in all patients with IBC (4).

The rarity of the disease makes conduction of specific, large prospective clinical trials more difficult. The objective of this study is to obtain high-quality evidence of the effectiveness of conserving surgery in IBC patients after NAC.

## Study design

The ConSIBreC trial is a prospective non-inferiority randomized study aimed at assessing the use of breast conserving surgery in patients with IBC that achieve clinical complete response after NAC.

Primary endpoint is local recurrence rate at 24 months after the date of surgery, and secondary endpoints are the rates of local recurrence-free survival and overall survival.

Diagnostic imaging with mammography, ultrasonography and breast MRI is necessary to detect the primary breast lesion and extension of disease. Detailed staging with PET/CT scans is required to determine the nodal status and distant metastases.

The diagnosis needs an histological characterization by needle biopsy of parenchymal lesion, a marker placement to identify the area of the breast tissue that will be removed, a drawing and a photography of the extent of the disease.

The study will include patients with IBC that achieve clinical complete response (proven by magnetic resonance, ultrasonography and mammogram) after NAC.

**Figure 1:**
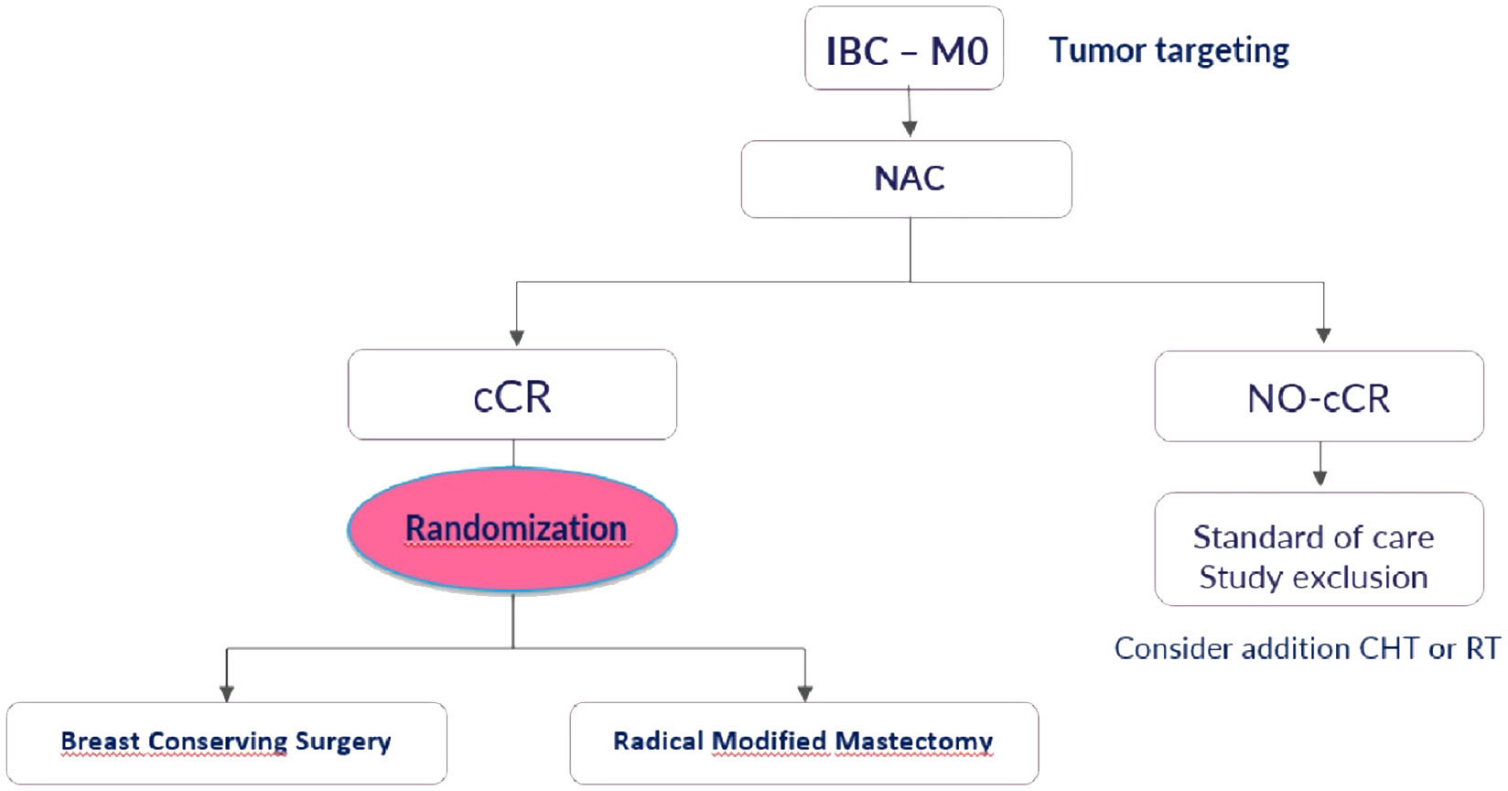
The ConSiBrec Trial: study design.

Patients fulfilling all eligibility criteria are randomly assigned (1:1 ratio) to either modified radical mastectomy (standard arm) or breast conserving surgery (experimental arm) (**Figure 1**).

All patients in both arms will receive adjuvant radiation therapy after surgery.

Data will be analyzed to evaluate statistical significance using Kaplan-Meier curves and log-rank tests.

Patient enrollment is planned to begin in January 2024 and will continue until a total of 329 patients per arm. The power analysis is carried out for two independent groups with a dichotomous primary outcome that is local recurrence rate. A minimum power level of 0.8 and a significance level of 0.05 are assumed.

Recruitment will adhere to strict inclusion criteria defined prior to the study protocol, including: patients affected by IBC with a clinical complete response after NAC; presence of a preinserted marker on target before NAC; written informed consent.

The exclusion criteria are: recurrent disease, metastatic patients, progression of disease or partial response after NAC.

Total study duration of 48 months (24 months inclusion, 24 months follow up).

## Discussion

In this era of surgical de-escalation several innovations in neoadjuvant systemic therapy have resulted in rising rates of clinical complete response in both the affected breast and the axilla. According to Nakhlis F. at al (8) there are very limited data on the safety of breast conservation of IBC; therefore, radical mastectomy without breast reconstruction remains the standard of care for this disease.

International consensus and guidelines still state that surgical treatment in IBC is a modified radical mastectomy regardless of response to NAC, breast conserving surgery is currently not recommended.

In a very recent meta-analysis of Lai et al. (9) the local recurrence rate for mastectomy and no surgery groups were 18.6% and 15.9%, respectively and no significant difference was observed between the two groups. Among patients with IBC who respond to NAC the local recurrence and 5-years survival rates in those undergoing breast conserving surgery are noninferior to the rates in those undergoing mastectomy; therefore, breast conserving surgery could be a feasible option for surgical management. However, we must consider that the sample of patients undergoing breast conserving surgery in the retrospective studies analyzed by the meta-analysis is very small.

There are some issues that should be considered: first of all, prospective randomized studies that have analyzed the difference in survival between conservative treatment and mastectomy after NAC have never included patients with IBC. Moreover, results that compared disease free survival and overall survival between breast conserving surgery and mastectomy in IBC are obtained exclusively from retrospective studies. Furthermore lack of uniformity in the diagnostic criteria, patient selection bias and different treatment regimens creates limitations in analyzing data retrospectively (10).

Due to these reasons, high level of evidence from randomized controlled trials is needed and the ConSIBreC trial was designed to fill this gap.

The ConSIBreC Trial was presented in October 2023 at 42nd Congress of the European Society of Surgical Oncology in Florence during the ESSO-EYSAC Dragon’s Den session supported by the Anticancer Fund and received the best clinical trial proposal award.

The European Society of Surgical Oncology (ESSO) has developed multiple clinical research programs as part of its mission to promote the best surgical care for cancer patients and to conduct innovative and high-quality surgical oncology research (11).

The young surgeon-scientist is an essential component of the field of academic surgery, contributing to the fundamental understanding of disease and the discovery of innovative therapies. Despite this the current landscape of academic medicine presents significant barriers to establishing and maintaining a successful career as a surgeon performing research (12).

The challenges facing young surgeon-scientists include increased competition for decreasing available funding, rising demand for clinical productivity, increasing complexity of the regulatory environment and changing expectations regarding work-life balance.

ESSO proposes a clear strategy based on 4 pillars to improve clinical research in surgery, including increasing funding and operational support in conducting research and aims to enable young surgeons to be active and establish partnerships for translational research (11).

## Conclusion

To date there are no prospective trials available in Literature studying the potential of conserving surgery in IBC. The ConSIBreC trial is the first randomized clinical trial that will provide real evidence about breast conserving surgery in IBC after NAC. Knowledge gain from this study will provide indications to consider surgical de-escalation as accetable in patients that achieve clinical complete response. The ConSIBreC trial is registered on ClinicalTrials.gov (NCT:06131632) and recruitment will start in January 2024.

## Author contributions

LS: Conceptualization, Investigation, Methodology, Resources, Validation, Visualization, Writing – original draft, Writing – review & editing. RM: Validation, Writing – review & editing. GF: Validation, Visualization, Writing – review & editing.
